# Genotyping Performance Assessment of Whole Genome Amplified DNA with Respect to Multiplexing Level of Assay and Its Period of Storage

**DOI:** 10.1371/journal.pone.0026119

**Published:** 2011-10-11

**Authors:** Daniel W. H. Ho, Wai Chi Yiu, Maurice K. H. Yap, Wai Yan Fung, Po Wah Ng, Shea Ping Yip

**Affiliations:** 1 Centre for Myopia Research, School of Optometry, The Hong Kong Polytechnic University, Hong Kong Special Administrative Region, China; 2 Department of Health Technology and Informatics, The Hong Kong Polytechnic University, Hong Kong Special Administrative Region, China; University of Texas MD Anderson Cancer Center, United States of America

## Abstract

Whole genome amplification can faithfully amplify genomic DNA (gDNA) with minimal bias and substantial genome coverage. Whole genome amplified DNA (wgaDNA) has been tested to be workable for high-throughput genotyping arrays. However, issues about whether wgaDNA would decrease genotyping performance at increasing multiplexing levels and whether the storage period of wgaDNA would reduce genotyping performance have not been examined. Using the Sequenom MassARRAY iPLEX Gold assays, we investigated 174 single nucleotide polymorphisms for 3 groups of matched samples: group 1 of 20 gDNA samples, group 2 of 20 freshly prepared wgaDNA samples, and group 3 of 20 stored wgaDNA samples that had been kept frozen at −70°C for 18 months. MassARRAY is a medium-throughput genotyping platform with reaction chemistry different from those of high-throughput genotyping arrays. The results showed that genotyping performance (efficiency and accuracy) of freshly prepared wgaDNA was similar to that of gDNA at various multiplexing levels (17-plex, 21-plex, 28-plex and 36-plex) of the MassARRAY assays. However, compared with gDNA or freshly prepared wgaDNA, stored wgaDNA was found to give diminished genotyping performance (efficiency and accuracy) due to potentially inferior quality. Consequently, no matter whether gDNA or wgaDNA was used, better genotyping efficiency would tend to have better genotyping accuracy.

## Introduction

With the availability of the complete sequence [1]–[3] and haplotype map [4]–[6] of the human genome, paradigm of genetic association studies has switched from candidate-gene design to genomewide approach. Linkage studies have been proven to be a successful strategy for Mendelian diseases with relatively low prevalence, high penetrance and large effect size. Nevertheless, efforts have been increasingly focused on common complex diseases, which are more appropriately and effectively tackled by association rather than linkage approach. With advancement in technology, genotyping of thousands to even millions of single nucleotide polymorphism (SNP) markers is now possible and widely available. This popularizes the genomewide association approach. No matter which approach, linkage vs association or candidate-gene vs genomewide, is adopted, a large number of genetic markers, most likely SNPs, have to be genotyped for a large number of subjects. Subject recruitment is always a major bottleneck for genetic studies. To recruit subjects for achieving enough statistical power, this step may take years to accomplish. The difficulty will be even greater for recruiting families.

One fundamental constraint on modern genetic studies is the limited supply of precious samples – genomic DNA (gDNA) extracted from blood in most cases. Despite the increasing level of multiplexing in genotyping and relatively small amounts of DNA required in most applications, the amount of gDNA extracted may still be insufficient for extensive use. Epstein-Barr virus-transformed cell lines have been used to provide unlimited amounts of DNA, but this method is labor-intensive, expensive and inapplicable to existing DNA samples. Several methods of whole genome amplification (WGA) [7] have also been developed to tackle this major challenge: PCR-based strategies using random oligonucleotide primers [8] or degenerate oligonucleotide primers [9], OmniPlex technology [10] and multiple displacement amplification (MDA) [11]. MDA is the most reliable method to faithfully amplify gDNA with minimal bias and substantial genome coverage [7], [12], [13]. It can generate products with average size >10 kb, and the relatively consistent product yield is less sensitive to the amount of starting material [7], [11]. Whole genome amplified DNA (wgaDNA) from MDA methods can be used in a variety of applications including high-throughput genotyping [7], [14], e.g., Affymetrix array [15] and Illumina BeadArray [16]. In addition, the starting DNA sample for WGA needs not to be fresh [17]. Such versatile applicability makes MDA the best and most popular WGA method. However, to our knowledge, it is not yet known whether wgaDNA samples would affect the overall performance with respect to multiplexing level (i.e. the complexity of assay), and whether the genotyping performance of wgaDNA would be affected by the period of storage.

This study is part of an on-going myopia genomics study. It provides comparative information on freshly prepared wgaDNAs and stored wgaDNAs (stored frozen for a period of time) against their gDNA counterparts. It also allows evaluation of the genotyping efficiency and accuracy for these three types of samples genotyped using the MassARRAY Sequenom SNP genotyping platform with iPLEX GOLD chemistry. Despite the fact that testing wgaDNA with the MassARRAY platform has been carried out before [17], the present study is the first one that systematically investigates the genotyping performance of wgaDNA with respect to the multiplexing level and the potential effect of storage period on wgaDNA genotyped using the same technology. Samples were amplified using MDA-based GenomiPhi V2 DNA Amplification Kit (GE Healthcare Life Sciences). MDA-based kit was used because of the numerous merits of the MDA method over others. Genotyping efficiency was assessed in terms of genotype completion rates while genotyping accuracy was evaluated based on genotype concordance rate between matched pairs of wgaDNA and gDNA samples. The effect of storage period on wgaDNA was also evaluated, and subgroup analysis stratified by multiplexing group was used to study the correlation of multiplexing level with wgaDNA usage.

## Results

### DNA quantification and quality control

gDNA samples were quantified using ultraviolet spectrophotometry and each sample had an initial concentration of >100 ng/µl. They were diluted to 15 ng/µl with Tris-EDTA (TE) solution. wgaDNA was amplified from 10 ng of gDNA according to the manufacturer's instruction. It has been found that at least 10 ng of gDNA should be used for WGA [12], [18]. The yield of wgaDNA ranged from ∼10 µg to 20 µg – an increase of at least 1000 times the starting amount of gDNA. They were diluted to 15 ng/µl with TE. A single SNP that had been successfully genotyped for gDNA samples by the method of restriction fragment length polymorphism as part of our on-going myopia genomics study was genotyped again for all wgaDNA samples by the same method. Samples that failed this quality control step were replaced. This served to ensure adequate quantity and good quality of all wgaDNA samples.

### Assessment of genotyping efficiency

We investigated the MassARRAY genotype data of 174 SNPs for the 3 groups of matched samples: group 1 (20 gDNA samples), group 2 (20 freshly prepared wgaDNA samples) and group 3 (20 stored wgaDNA samples that had been stored frozen at −70°C for 18 months). To be consistent and precise, we hereafter used the term “stored wgaDNA” to refer specifically to wgaDNA that had been stored frozen at −70°C for 18 months, unless stated otherwise.

**Figure 1:**
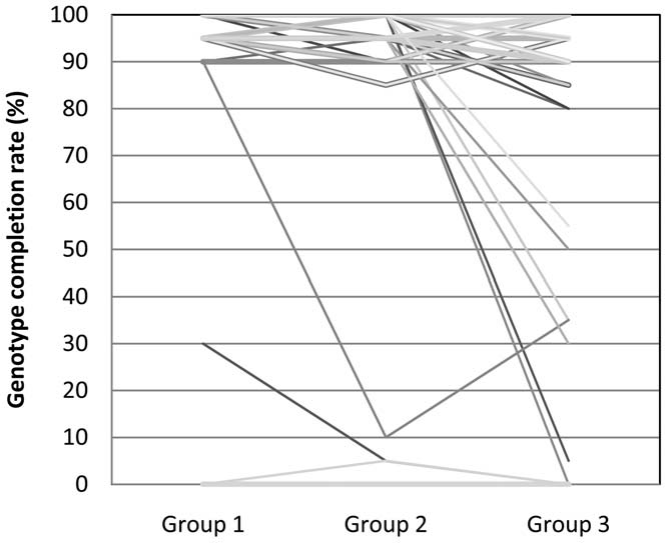
Genotyping efficiency for different sample groups based on 174 SNPs genotyped using MassARRAY assay.

We used genotype completion rates to assess genotyping efficiency. The mean genotype completion rates for groups 1 to 3 samples were 96.8%, 96.2% and 93.0%, respectively (Table 1). Obviously, group 1 gDNA samples achieved the highest mean genotype completion rate for the 174 SNPs genotyped and the values were also the least dispersed with the lowest SD of 12.0% (Figure 1).

**Table 1:** Summary of genotyping efficiency among sample groups stratified by multiplexing level.

		Mean genotype completion rate (SD)				*P* value for pairwise comparison of mean genotype completion rates (Group a vs b)^e^		
Multiplexing level	No. of SNPs	Group 1^a^	Group 2^b^	Group 3^c^	*P* value^d^	1 vs 2	1 vs 3	2 vs 3
All	174	96.8 (12.0)	96.2 (14.4)	93.0 (19.0)	**0.002**	0.431	**1.73e-04**	**0.003**
17-plex	17	99.1 (2.0)	99.4 (1.7)	93.8 (6.3)	**1.12e-04**	0.317	**0.007**	**0.005**
21-plex	21	95.7 (15.2)	94.8 (20.6)	91.0 (25.1)	0.186	0.655	0.161	0.096
28-plex	28	98.6 (2.3)	98.2 (2.8)	97.5 (5.0)	0.723	0.480	0.305	0.417
36-plex	108	96.2 (13.5)	95.4 (15.7)	92.2 (21.1)	0.269	0.557	**0.036**	0.211

a Mean genotype completion rates were not significantly different (*P* = 0.197, Kruskal-Wallis test) across multiplexing levels. However, there was significant, albeit weak, correlation between mean genotype completion rates and multiplexing levels (coefficient = −0.163, *P* = 0.032).

b Mean genotype completion rates were not significantly different (*P* = 0.168, Kruskal-Wallis test) across multiplexing levels. However, there was significant, albeit weak, correlation between mean genotype completion rates and multiplexing levels (coefficient = −0.155, *P* = 0.041).

c Mean genotype completion rates was not significantly different (*P* = 0.078, Kruskal-Wallis test) across multiplexing levels. There was no significant correlation between mean genotype completion rates and multiplexing levels either (coefficient = 0.045, *P* = 0.599).

d *P* values for comparison of mean genotype completion rates across three matched sample groups by Friedman test.

e *P* values for matched pairwise comparison of mean genotype completion rates by Wilcoxon signed rank test.

To find out whether the use of wgaDNA samples and the storage period of wgaDNA would reduce the genotyping efficiency, mean genotype completion rates were compared among these 3 sample groups (Table 1) and found to be significantly different (*P* = 0.002). As expected, pairwise comparison did not show any significant difference in mean genotype completion rate between groups 1 and 2 (*P* = 0.431, Table 1). However, significant differences were shown between groups 1 and 3 (*P* = 1.73e-4) and between groups 2 and 3 (*P* = 0.003). This indicated that the storage period of wgaDNA was likely to reduce the genotyping efficiency.

Stratified analyses were performed to investigate the potential effect of using stored wgaDNA on the genotyping efficiency with respect to multiplexing level in the MassARRAY assay. Again, considering mean genotype completion rate *across* sample groups, there were significant differences only at the 17-plex level (*P* = 1.12e-4; Table 1). On the other hand, *within* each sample group, there was no significant difference in mean genotype completion rates among four multiplexing levels (*P* = 0.197, 0.168, 0.078 for groups 1 to 3 respectively; footnotes a to c, Table 1). When genotype completion rates were compared across multiplexing levels (17-plex, 21-plex, 28-plex and 36-plex) for potential *correlation*, significant albeit weak correlation could be detected for groups 1 and 2 samples, but not group 3 samples.

For pairwise comparisons stratified by multiplexing level, the most contrasting differences were detected between groups 1 and 3 samples although there were no significant differences at certain multiplexing levels (all: 1.73e-04; 17-plex: *P* = 0.007; 21-plex: *P* = 0.161; 28-plex: *P* = 0.305; 36-plex: *P* = 0.036; Table 1). Similar results were detected between groups 2 and 3 samples (all: 0.003; 17-plex: *P* = 0.005; 21-plex: *P* = 0.096; 28-plex: *P* = 0.417; 36-plex: *P* = 0.211; Table 1). No obvious trend could be detected with these stratified pairwise comparisons.

### Assessment of genotyping accuracy

Genotyping accuracy was measured by means of genotype concordance rate between pairs of sample groups for all 174 SNPs genotyped (Table 2). The mean genotype concordance rates were 97.9% (SD, 6.9%) between groups 1 and 2 samples, 96.9% (SD, 6.9%) between groups 1 and 3 samples, and 93.6% (SD, 14.5%) between groups 2 and 3 samples. The overall difference in the mean genotype concordance rates for all pairs of sample groups was statistically significant (*P* = 8.47e-9) (Table 2).

**Table 2:** Summary of genotyping accuracy among sample groups stratified by multiplexing level.

		Mean genotype concordance rate (SD) between groups a & b				*P* value for pairwise comparison of mean genotype concordance rates [(Groups a & b) vs (Groups c & d)]^e^		
Multiplexing level	No. of SNPs	1 & 2^a^	1 & 3^b^	2 & 3^c^	*P* value^d^	(1 & 2) vs (1 & 3)	(1 & 2) vs (2 & 3)	(1 & 3) vs (2 & 3)
All	174	97.9 (6.9)	96.9 (6.9)	93.6 (14.5)	**8.47e-09**	**0.039**	**5.81e-08**	**2.84e-06**
17-plex	17	99.1 (2.0)	92.9 (9.2)	91.5 (11.4)	**0.002**	**0.011**	**0.007**	0.726
21-plex	21	97.6 (6.3)	95.5 (11.8)	93.8 (16.5)	0.42	0.378	0.523	0.614
28-plex	28	95.4 (15.0)	98.0 (3.9)	96.4 (5.1)	0.076	0.805	0.238	**0.020**
36-plex	108	98.4 (3.0)	97.5 (5.5)	93.1 (16.2)	**3.18e-07**	0.274	**7.75e-07**	**9.14e-06**

a Mean genotype concordance rates was not significantly different (*P* = 0.491, Kruskal-Wallis test) across multiplexing levels. In addition, there was no significant correlation between mean genotype concordance rates and multiplexing levels (coefficient = -0.025, *P* = 0.746).

b Mean genotype concordance rates was marginally different (*P* = 0.050, Kruskal-Wallis test) across multiplexing levels. In addition, there was significant correlation between mean genotype concordance rates and multiplexing levels (coefficient = 0.155, *P* = 0.041).

c Mean genotype concordance rates was not significantly different (*P* = 0.184, Kruskal-Wallis test) across multiplexing levels. In addition, there was no significant correlation between mean genotype concordance rates and multiplexing levels (coefficient = -0.008, *P* = 0.917).

d *P* values for comparison of mean genotype concordance rates by Friedman test.

e *P* values for matched pairwise comparison of mean genotype concordance rates by Wilcoxon signed rank test.

For groups 1 and 2, there was neither significant difference in the mean genotype concordance rate among different multiplexing levels (*P* = 0.491) nor significant correlation between mean genotype concordance rate and multiplexing level (coefficient = -0.025, *P* = 0.746; footnote a, Table 2). The same was true for groups 2 and 3: no significant difference in the mean genotype concordance rate among different multiplexing levels (*P* = 0.184), and no significant correlation between mean genotype concordance rate and multiplexing level either (coefficient = -0.008, *P* = 0.917; footnote c, Table 2). On the other hand, for groups 1 and 3, there was marginally significant difference in the mean genotype concordance rate among different multiplexing levels (*P* = 0.050), and significant correlation could be identified between mean genotype concordance rate and multiplexing level (coefficient = 0.155, *P* = 0.041; footnote b, Table 2). In addition to genotype concordance rate, evidence for high degree of genotype agreement also came from the kappa statistics over the 174 SNPs (3480 possible genotype pairs): 0.976 for groups 1 and 2, 0.964 for groups 1 and 3, and 0.927 for groups 2 and 3 (details not shown).

The majority of discordant genotype pairs were due to having genotype in one sample while having no genotype call in another of the pair: 62 out of the 72 discordant genotype pairs between groups 1 and 2, 104 out of 111 discordant genotype pairs between groups 1 and 3, and 194 out of 223 discordant genotype pairs between groups 2 and 3. There were more missing calls for stored wgaDNA (group 3). For discordant genotype pairs having genotype calls from both samples (i.e. no missing genotype call), all involved having heterozygous genotype in one sample (either gDNA or wgaDNA) while having homozygous genotype in another (either gDNA or wgaDNA). These included 10 out of 72 discordant genotype pairs between groups 1 and 2, 7 out of 111 discordant pairs between groups 1 and 3, and 29 out of 223 discordant pairs between groups 2 and 3. However, no special pattern could be detected because equal numbers of heterozygous-to-homozygous or homozygous-to-heterozygous genotype discrepancies were observed. In addition, variables such as the type of polymorphisms (A/C, A/G, A/T, C/G, C/T or G/T), the GC content of PCR products, the extension primer and the length of PCR products were also examined, but found to have no correlation with the genotype concordance rate.

### Correlation between genotyping efficiency and accuracy

For groups 1 and 2 samples, their genotype *completion rates* were correlated with the genotype *concordance rate* between them: coefficient = 0.375, *P* = 3.36e-7 for group 1 samples; and coefficient = 0.303, *P* = 4.83e-5 for group 2 samples (Table 3). In other words, significant and positive correlation could be detected with both sample groups, and this probably indicated that better genotyping efficiency led to better genotyping accuracy. Similarly, for groups 1 and 3 samples, significant correlation could also be found between their genotype completion rates and the genotype concordance rate between them (coefficient = 0.323, *P* = 1.34e-5 for group 1 samples; and coefficient = 0.588, *P* = 1.47e-17 for group 3 samples; Table 3). For groups 2 and 3 samples, the correlation between their genotype completion rates and the genotype concordance rate between them was even stronger and more significant: coefficient = 0.349, *P* = 2.28e-6 for group 2 samples; and coefficient = 0.624, *P* = 3.59e-20 for group 3 samples (Table 3). This overall correlation also generally matched the stratified correlation except at certain multiplexing levels (Table 3). Stratified analysis indicated that this correlation was more consistently detected, but not necessarily stronger in magnitude, at higher level of multiplexing than lower level of multiplexing: 6 out of 6 scenarios for 36-plex assays, but only 2 out of 6 scenarios for 17-plex assay. This could probably be explained by the relatively smaller sample size for the 17-plex level with only 17 SNPs when compared with 108 SNPs for the 36-plex level.

**Table 3:** Summary of correlation between genotyping efficiency and accuracy among groups 1, 2 and 3 samples stratified by multiplexing level.

		Correlation between completion rate and concordance rate for genotypes (groups 1 vs 2)		Correlation between completion rate and concordance rate for genotypes (groups 1 vs 3)		Correlation between completion rate and concordance rate for genotypes (groups 2 vs 3)	
Multiplexing level	No. of SNPs	Group 1	Group 2	Group 1	Group 3	Group 2	Group 3
All	174	**0.375 (** ***P*** ** = 3.36e-7)**	**0.303 (** ***P*** ** = 4.83e-5)**	**0.323 (** ***P*** ** = 1.34e-5)**	**0.588 (** ***P*** ** = 1.47e-17)**	**0.349 (** ***P*** ** = 2.28e-6)**	**0.624 (** ***P*** ** = 3.59e-20)**
17-plex	17	0.190 (*P* = 0.464)	-0.169 (*P* = 0.517)	0.140 (*P* = 0.592)	**0.884 (** ***P*** ** = 2.54e-6)**	-0.097 (*P* = 0.710)	**0.838 (** ***P*** ** = 2.70e-5)**
21-plex	21	**0.638 (** ***P*** ** = 0.002)**	**0.471 (** ***P*** ** = 0.031)**	**0.536 (** ***P*** ** = 0.012)**	0.073 (*P* = 0.755)	0.142 (*P* = 0.540)	**0.583 (** ***P*** ** = 0.006)**
28-plex	28	0.342 (*P* = 0.075)	**0.431 (** ***P*** ** = 0.022)**	0.205 (*P* = 0.296)	**0.789 (** ***P*** ** = 5.97e-7)**	**0.683 (** ***P*** ** = 6.18e-5)**	**0.666 (** ***P*** ** = 1.08e-4)**
36-plex	108	**0.359 (** ***P*** ** = 1.35e-4)**	**0.275 (** ***P*** ** = 0.004)**	**0.329 (** ***P*** ** = 0.001)**	**0.561 (** ***P*** ** = 2.59e-10)**	**0.392 (** ***P*** ** = 2.77e-5)**	**0.556 (** ***P*** ** = 4.07e-10)**

## Discussion

Consistent with previous studies, the present study showed that wgaDNAs (freshly prepared or stored at −70°C for 18 months) had satisfactory genotyping efficiency collectively and at various multiplexing levels (Table 1). In addition, the high concordance of genotypes between group 1 (gDNA) and group 2 (fresh wgaDNA) samples as well as between group 1 (gDNA) and group 3 (stored wgaDNA) samples indicated the high genotyping accuracy of wgaDNA (Table 2). This further testified the validity of using wgaDNA as a replacement for gDNA. More importantly, together with the findings from other wgaDNA studies, the present study highlighted the great scalability of our existing limited gDNA assets. In other words, the application of WGA on gDNA expands the amount of our valuable DNA samples such that they can last for more experiments. Since the WGA process can normally amplify DNA by >1000-fold, it is anticipated that the samples can last for substantially greater number of use.

The validity of wgaDNA for ordinary use has been well justified [7], [14] and wgaDNA has previously been tested on medium-throughput MassARRAY platform with iPLEX GOLD chemistry [17]. However, to our knowledge, this study is the first one that made use of the variable multiplexing ability of the same genotyping technology to study the relationship between genotyping performance and multiplexing level of the assays using wgaDNA, and to examine whether the storage of wgaDNA would reduce the genotyping efficiency. Indeed, existing studies [15], [19]–[22] have successfully addressed the question of whether wgaDNA could be used in high-throughput array-based assays, e.g., Affymetrix and Illumina genotyping chips. High concordance was detected between wgaDNA and gDNA, highlighting the reliability of using wgaDNA for high-throughput genotyping with good accuracy. Nonetheless, different assays have different reaction chemistries. Such successful application of wgaDNA in high-throughput array-based assays does not necessarily imply that genotyping performance would not deteriorate with increasing multiplexing level of the assays upon the use of wgaDNA. While high-throughput array-based assays use either uniform or random primers for template amplification, MassARRAY assays employ a different technology that requires multiple sets of specific primers to amplify multiple specific regions. Such specificity requirement becomes even more critical with increasing level of multiplexing in the MassARRAY assays. From this perspective, results from previous studies of high-throughput array-based assays [15], [16] or even the MassARRAY-based study of a single multiplex group of 35-plex by Hollegaard et al. [17] could not be extrapolated directly to provide the necessary information on the issues addressed by our current study. This was the reason why our current study was carried out.

In our study, data were stratified according to sample group and multiplexing level. Analysis of the **mean genotype completion rates** among the 3 sample groups detected statistically significant difference (*P* = 0.002) (Table 1), suggesting that there was a marked difference. Indeed, by pairwise comparison of mean genotype completion rates (Table 1), groups 1 and 3 samples were found to show the most remarkable difference in genotyping efficiencies (*P* = 1.73e-4; group 3 being lower than group 1, 93.0% vs 96.8%). Similarly, difference was also found between genotyping efficiencies of groups 2 and 3 samples (*P* = 0.003; group 3 being lower than group 2, 93.0% vs 96.2%). This evidence suggested the inferior quality of group 3 samples because group 2 (freshly prepared wgaDNA) samples were found to have similar genotyping efficiency as group 1 (gDNA) samples.

Moreover, mean genotype completion rates of SNPs were compared *across* different multiplexing levels (Table 1). Although the mean genotype completion rates seemed to be different among the groups by visual inspection, the difference was not statistically significant in any sample group because of the high variance of genotype completion rates (footnotes a to c, Table 1). However, significant *correlation*, despite quite weak, could be detected between genotype completion rates and multiplexing levels in sample groups 1 and 2 (footnotes a and b, Table 1). This suggested that genotype completion rate decreased monotonically with increasing multiplexing level in these 3 groups of samples. It is intuitive to think that the complexity of the assay increases with increasing level of multiplexing, and this poses greater difficulty to the assay and adversely affects the genotyping efficiency. That the correlation was weak could probably reflect the effectiveness of the primer design algorithm used by the Sequenom assays for multiplex PCR. Multiplexing that has a high chance of failure might have been removed beforehand. Other factors like the type of genetic polymorphisms tested also influence the overall complexity of the assay. This phenomenon seemed to be valid in sample groups 1 and 2, but not 3. Group 3 samples were stored wgaDNAs that had been kept at −70°C for 18 months.

Our result showed that the overall genotype completion rate of group 3 samples (93.0%) was the lowest among the 3 sample groups (Table 1). Given that groups 2 (freshly prepared wgaDNA) and 3 (stored wgaDNA) samples were both originated from the same WGA reactions (2 sets of aliquots of the same WGA products and immediately frozen at −70°C after completing the WGA), the major difference between them was the storage period. Group 3 samples had been stored for 18 months while group 2 samples were used for genotyping within one week after WGA. The quality of group 3 samples could be inferior to that of the others such that the potential correlation between genotyping efficiency and multiplexing level could not be observed (footnote c, Table 1). Our data suggested that, with gDNAs as the reference, the correlation was not disrupted or exaggerated by the use of freshly prepared wgaDNA samples, which were believed to have better quality than stored wgaDNA samples. This indicated that the use of freshly prepared wgaDNA would not incur additional adverse burden although increasing multiplexing level was suggested to lead to slight deterioration in genotyping efficiency.

On the other hand, concerning genotyping accuracy, high concordance of genotypes could be observed between groups 1 and 2, groups 1 and 3 as well as groups 2 and 3 samples in terms of genotype concordance rate (Table 2) and kappa statistic (details not shown). Nonetheless, significant difference could be detected among the mean **genotype concordance rates** (*P* = 8.47e-9, Table 2) with that between groups 2 and 3 samples being the lowest (93.6%). As a result, the use of stored wgaDNA was likely to reduce the genotyping accuracy of MassARRAY assays. If group 1 gDNA samples are treated as the reference, the mean genotype concordance rates with group 1 samples will reflect the variability or uncertainty in genotype accuracy – the lower the genotype concordance rate, the more uncertain it is for the genotyping results. Indeed, the degree of uncertainty was significantly higher for group 3 samples. As there were some discrepancies between groups 1 and 2 samples, it was reasonable to believe that even freshly prepared wgaDNA samples could introduce some degree of uncertainty to the genotyping results due to the WGA process. More importantly, the mean genotype concordance rate was even worse between groups 1 and 3 samples. This indicated that 18-month storage of wgaDNA samples even at −70°C further deteriorated the situation by introducing additional uncertainty to the genotyping results. Therefore, the concordance was the lowest (93.6%, Table 2) between group 2 (with uncertainty from WGA process) and group 3 (with uncertainties from both the WGA process and the storage period) samples. Taken together, our results showed that the genotyping performance of freshly prepared wgaDNA, but not stored wgaDNA, was similar to that of gDNA. Therefore, this evidence highlighted the importance of using fresh wgaDNA samples in order to obtain better genotyping performance for MassARRAY assays.

Since MassARRAY assay is a popular medium-throughput genotyping platform for following up a moderate number of SNP markers, researchers might enjoy the high scalability of existing DNA samples by using WGA on one hand, and relieve their worries about potential burden of wgaDNA on variable multiplexing assay on the other hand. As stored wgaDNAs were suggested to have inferior genotyping efficiency and accuracy, further study is warranted to investigate whether stored wgaDNAs would have lower genotyping performance in other genotyping platforms.

There were limitations in our current study. First, the scale of our study is relatively small with only 60 matched samples (20 gDNA, 20 freshly prepared wgaDNA and 20 stored wgaDNA). Second, our current study has not yet addressed in depth the effect of long-term storage on the quality of wgaDNA samples because group 3 (stored wgaDNA) samples have only been stored for 18 months before use. Third, our current study did not include a clean-up step after WGA.

Given our experience to date with wgaDNA stored frozen at −70°C for up to 18 months, further study of a larger sample set stored for even longer periods (at least 2–5 years or longer) would be warranted to confirm our initial findings of its deleterious effects on genotyping efficiency and accuracy. Based on our results of reduced genotyping performance with increasing storage time, especially in terms of accuracy, caution is indicated for genotyping data of wgaDNA samples. Cautious handling of wgaDNA is also important. First, it is necessary to have better planning for experiments so as to minimize the storage period for wgaDNA. Second, making reasonable aliquots of wgaDNA samples can effectively reduce the number of freezing-thawing cycles, which is believed to seriously influence the quality of all DNA samples including wgaDNA. Third, WGA products should be purified and kept in TE buffer instead of water. It is of particular concern that Mg^2+^ ion is present in the WGA reaction buffer and is required for the enzymatic activity of the ϕ29 DNA polymerase used in MDA. This metal ion is also the co-factor for DNase, an enzyme degrading DNA. It may thus be a good idea to remove it by purification after WGA. It might also be good to report the storage period for wgaDNA in publications because variation of genotyping efficiency in different studies could be due to different storage periods of wgaDNA samples.

Last but not least, our results suggested that the higher the genotyping efficiency, the better the genotyping accuracy was (Table 3). This relationship was valid for all gDNA, freshly prepared wgaDNA and stored wgaDNA. This suggests that MassARRAY assay is reliable for all kinds of samples when the genotyping efficiency (i.e. genotype completion rate) is high.

In summary, significant, though weak, correlation between genotyping efficiency and multiplexing level was detected in both gDNA and freshly prepared wgaDNA. Since the degree of correlation was similar for both sample groups, this indicated the absence of *additional* adverse effect of using freshly prepared wgaDNA on genotyping efficiency of MassARRAY assay although increasing multiplexing level tended to lead to modest deterioration in genotyping efficiency in general. Moreover, the genotyping performance of freshly prepared wgaDNAs was found to be similar to that of gDNA collectively and with respect to various multiplexing levels. However, stored wgaDNA gave lower genotyping efficiency and accuracy than gDNA and freshly prepared wgaDNA due to potentially inferior quality. Finally, there was a significant correlation between genotyping efficiency and genotyping accuracy. Therefore, MassARRAY assay is reliable when genotyping efficiency is satisfactory.

## Materials and Methods

### DNA Samples

For the purpose of performance comparison, three groups of matched DNA samples were used in this study: 20 gDNA samples extracted from whole blood (group 1), 20 wgaDNA samples freshly amplified from the corresponding “group 1” gDNA (group 2) and 20 wgaDNA samples amplified from the corresponding "group 1" gDNA and stored frozen at −70°C for 18 months (group 3). Group 2 and 3 samples were aliquots of the same WGA products, which were prepared using GenomiPhi V2 DNA Amplification Kit (GE Healthcare Life Sciences) according to the manufacturer's instructions, and used for genotyping without further purification. Group 2 samples were freshly prepared and used for genotyping within one week after WGA. Group 3 samples had been stored at −70°C for 18 months prior to the genotyping process. Both groups of wgaDNA samples were immediately frozen after the WGA process, and thawed before use to avoid repeated freezing-thawing cycles. Informed consent was obtained from all subjects. Ethical approval for the study was obtained from the Human Subjects Ethics Subcommittee of the Hong Kong Polytechnic University, and adhered to the tenets of the Declaration of Helsinki.

### SNP genotyping

Genotyping of 174 SNPs was done at the Genome Research Centre, the University of Hong Kong, as a contract service using the Sequenom MassARRAY technology platform with the iPLEX GOLD chemistry (Sequenom, San Diego, CA). The manufacturer's protocols were followed closely. Briefly, specific assays were designed using MassARRAY AssayDesign software package (v3.1) with filtering of proximal SNPs and checking of specificity for PCR amplification and the subsequent primer extension reaction. One µl of DNA sample (15 ng/µl) was used in each PCR. Residual nucleotides were dephosphorylated before the iPLEX GOLD reaction. After single-base extension, reaction products were desalted with SpectroCLEAN resin (Sequenom, San Diego, CA), and an aliquot of 10 nL of the desalted product was spotted onto a 384-format SpectroCHIP with the MassARRAY Nanodispenser. Mass determination was done with the MassARRAY Analyzer Compact MALDI-TOF mass spectrometer. The MassARRAY Typer 4.0 software was used for data acquisition and analysis. Genotypes were called after cluster analysis using the default setting of Gaussian mixture model. Genotype calls were then further reviewed manually to undo any uncertain calls due to clustering artifact. Assay with less than 80% call rate within the same SpectroCHIP was considered failed. For every 96-well sample plate, one well was used for blank control and five wells for duplicate check. SpectroCHIP with more than 25% call rate in the blank control was considered failed and would be repeated. SpectroCHIP with less than 99.5% concordance in duplicate checks along with more than 10% call rate in blank check was also considered failed.

### Statistical Analysis

To measure genotyping efficiency, means and variances of genotype completion rates (percentage of successful genotype calls, i.e. proportion of samples that could be genotyped successfully with respect to an individual SNP as well as overall dataset) were compared among 3 groups of samples by nonparametric Wilcoxon signed rank test, Friedman test and Kruskal-Wallis test as appropriate. Genotyping accuracy was evaluated by pairwise comparison of actual genotypes between matched sample pairs (groups 1 and 2, groups 1 and 3 as well as groups 2 and 3), and agreement was summarized in terms of genotype concordance rate (percentage of identical genotype calls) and kappa statistic. Data were stratified by multiplexing level in MassARRAY assay with groups of SNPs multiplexed in 17-plex (17 SNPs), 21-plex (21 SNPs), 28-plex (28 SNPs) and 36-plex (3 sets; 108 SNPs in total). Nonparametric Spearman correlation was used to detect correlation between variables. Analysis was done with SPSS (ver. 16.0, Chicago, IL) and Excel.
